# Similar degrees of obesity induced by diet or aging cause strikingly different immunologic and metabolic outcomes

**DOI:** 10.14814/phy2.12708

**Published:** 2016-03-31

**Authors:** Kanthi B. Krishna, Maja Stefanovic‐Racic, Nikolaos Dedousis, Ian Sipula, Robert M. O'Doherty

**Affiliations:** ^1^ Division of Pediatric EndocrinologyChildren's Hospital of Pittsburgh of UPMCPittsburghPennsylvania; ^2^ Division of Endocrinology and MetabolismDepartment of MedicineUniversity of PittsburghPittsburghPennsylvania

**Keywords:** Aging, immunology, obesity

## Abstract

In obesity, adipose tissue (AT) and liver are infiltrated with Th‐1 polarized immune cells, which are proposed to play an important role in the pathogenesis of the metabolic abnormalities of obesity. Aging is also associated with increased adiposity, but the effects of this increase on inflammation and associated metabolic dysfunction are poorly understood. To address this issue, we assessed insulin resistance (IR) and AT and liver immunophenotype in aged, lean (AL) and aged, obese (AO) mice, all of whom were maintained on a standard chow diet (11% fat diet) throughout their lives. For comparison, these variables were also assessed in young, lean (YL) and young diet‐induced obese mice (41% fat diet, YO). Despite similar body weight and fat accumulation, YO mice were substantially more IR and had greater liver steatosis compared to AO mice. YO also had elevated infiltration of macrophages/dendritic cells in AT and liver, but these increases were absent in AO. Furthermore, liver immune cells of YO were more Th‐1 polarized then AO. Notably, aging was associated with accumulation of T cells, but this occurred independent of obesity. Together, the data suggest that reduced inflammation in AO underlies the improved insulin sensitivity and lowered steatosis compared to YO.

## Introduction

Human and rodent obesity are associated with a state of chronic low‐grade inflammation, including myeloid and lymphoid cell infiltration of adipose tissue (AT) and liver. In the last decade it has become clear that these tissue immunophenotype alterations play an important role in the pathogenesis and exacerbation of the metabolic abnormalities of obesity, most notably insulin resistance, steatosis, and generalized dyslipidemia (Olefsky and Glass 2010; Gregor and Hotamisligil 2011; Lumeng and Saltiel 2011; Osborn and Olefsky 2012). Aging in humans and rodents is also associated with increased adiposity, which has been proposed to result from some combination of changes in metabolic rate, decreased activity, and loss of muscle mass (Villareal et al. 2005; Maggi et al. 2006; Morley and Sinclair 2009; Barzilai et al. 2012; Flegal et al. 2012; Kalyani and Egan 2013; Kohara 2014). Furthermore, there is substantial evidence of altered inflammatory status in aging (Jensen 2008; Ahima 2009; Lumeng et al. 2011; Garg et al. 2014; Feldman et al. 2015). However, to what extent aging‐related increases in adiposity facilitate tissue inflammation and metabolic alterations similar to those observed in the more commonly studied models of diet‐induced or genetic obesity remains largely unstudied. To address this issue, we have taken advantage of a serendipitous observation that cohorts of aged (~18 months) C57Bl/6J mice spontaneously separated into two distinctive body types (lean and obese), and compared the immune and metabolic phenotypes of these aged mice to those of young lean (YL) mice and young mice rendered obese by feeding a high‐fat diet. The data demonstrate that diet‐induced obesity has a profoundly greater impact on metabolic homeostasis compared to aging‐induced obesity. Importantly, the less impaired metabolic profile in aging‐associated obesity is associated with the absence of the AT and liver inflammatory responses that occur in diet‐induced obesity.

## Materials and Methods

### Animals and diet

Three‐week‐old male C57Bl/6J mice were purchased from The Jackson Laboratory (Bar Harbor, ME) and housed at the University of Pittsburgh animal facility with ad libitum access to food and water. Upon arrival, the mice were immediately exposed to either a standard chow diet (SCD; 65% carbohydrate, 11% fat, 24% protein per calories) or a test high‐fat diet (HFD; 40% carbohydrate, 41% fat, 19% protein per calories; Harlan Teklad, Madison, WI) for 14 weeks (YL and young obese [YO], respectively). For the aged group, mice were exposed to the SCD diet only, for 14–15 months throughout life (18 months). In preliminary experiments, a comparison of a low‐fat test diet and the SCD used in this study found no differences in weight gain, adiposity, or the metabolic and immunophenotypic variables assessed here (data not shown). At 16 months of age, these mice were categorized based on their degree of adiposity as aged lean (AL, fat mass <9.5 g) or aged obese (AO, fat mass >10.5 g). Based on weight checks of a cohort of mice between 12 and 15 months of age, there was no crossover from the lean to obese phenotype or vice versa (data not shown). All experiments were conducted in compliance with the National Institute of Health guidelines and all procedures were approved by the University of Pittsburgh Institutional Animal Care and Use Committee.

### Experimental design

At the age of 17 weeks (young group) or 16 months (aged group), body composition was assessed by Echo MRI (Houston, TX). Metabolic rate and activity were assessed using the CLAMS system (Comprehensive Laboratory Animal Monitoring System, Columbus Instruments, Columbus, OH). Intraperitoneal glucose tolerance tests (IP GTT) were performed by injecting 6‐h fasted mice with 1.5 g/kg of glucose in 200 μL of saline. Blood glucose levels were obtained at baseline and every 15 min for a total of 135 min via tail vein sampling (Contour Glucose Meter, Bayer Health Care, Mishawaka, IN). In separate experiments, mice were injected IP with 1.5 g/kg glucose. After 30 min (i.e., at peak plasma glucose levels in GTT), blood was drawn via cardiac puncture and plasma glucose and insulin values were assessed. Harvested tissues were either used for isolation of immune cells or flash frozen at −80°C for further analysis.

### Flow cytometry

Mononuclear cells from liver and spleen and stromal vascular cells (SVC) from AT (epididymal and perirenal depots) were isolated as described previously (Stefanovic‐Racic et al. 2012). Cell suspensions (2 × 10^6^ cells/sample) were preincubated with anti‐CD16/32 (Fc “blocking” antibodies [Abs]) for 15 min at 4°C, then stained with either fluorescent‐labeled Abs or IgG isotype controls for 30 min at 4°C. The following Abs were used: CD8/PerCP and CD45/PerCP (BD Biosciences, San Jose, CA), CD4/FITC, CD62L/PE, NK1.1/PeCy7, B220/v450, CD3/APC, CD86/FITC, MHC2/PE, CD11b/PeCy7, B220/v450, CD11c/APC, and F4/80/Alexa780 (eBiosciences, San Diego, CA). Following incubation with Abs, liver and AT cells were incubated in Aquaporin dye (Invitrogen, Grand Island, NY) for 15 min, to distinguish live from dead cells, then fixed in 4% paraformaldehyde (Fisher, Waltham, MA) before being analyzed using a FACSCalibur flow cytometer and FACSDiva software (BD Biosciences). A proportion of up to 1% false‐positive events were accepted in the isotype control samples.

### Tissue and plasma measurements

The isolated immune cells from liver and SVC from AT were cultured for 18 h in complete DMEM medium (Cellgro, Manassas, VA). The harvested media was analyzed for various cytokines (TNF‐α, INF‐γ, IL‐6, MCP‐1, IL‐4, IL‐13, IL‐10) using Luminex (Invitrogen). Liver triglyceride (TG) levels were measured using commercially available kit (Infinity Triglycerides, Thermo Scientific, Middletown, VA) (Stefanovic‐Racic et al. 2008) and plasma insulin levels were measured by ELISA kit (Crystal Chem. INC, Downers Grove, IL) according to the manufacturer's protocol.

### Statistical analysis

Results are expressed as mean ± SEM for 4–12 animals in each group. Data were analyzed by either the two‐tailed Student's *t*‐test or ANOVA (repeated measures or one‐way, followed by the Tukey's post hoc test) using the SPSS 21.0 software, where appropriate. Significance was set at *P *<* *0.05.

## Results

### Metabolic characteristics of YL, YO, AL, and AO mice

**Figure 1:**
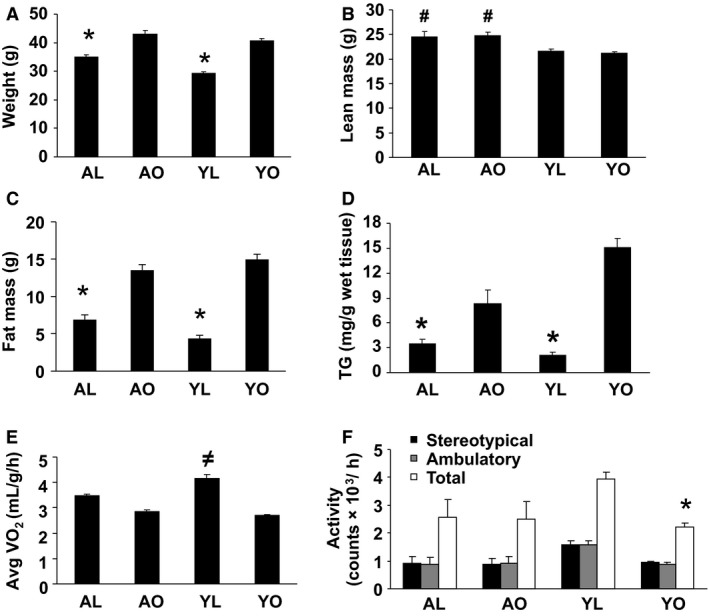
Physiological characteristics of mice. Young lean (YL) and young obese (YO) 3‐week‐old C57Bl6/J mice were exposed to either a standard chow (YL) or high‐fat (YO) diet for 14 weeks prior to analysis. Aged lean (AL) and aged obese (AO) C57Bl6/J mice were exposed to a standard chow diet for 14–15 months prior to analysis. Mice were then separated into AL or AO based on their degree of adiposity (AL, fat mass <9.5 g, AO fat mass >10.5 g). A minimum of six animals per group were individually analyzed and results are presented as means ± SE. Significant differences are indicated (*as compared to the other three groups, ^#^as compared to YL and YO, ^≠^as compared to YO and AO, *P* < 0.05). (A) Animal weights. (B and C) Fat mass and lean mass obtained via echo MRI at 17 weeks (young) or 16 months (aged) of age. (D) Triglyceride (TG) content of the liver. (E) Energy expenditure assessed by CLAMS. (F) Activity assessed by CLAMS.

**Figure 2:**
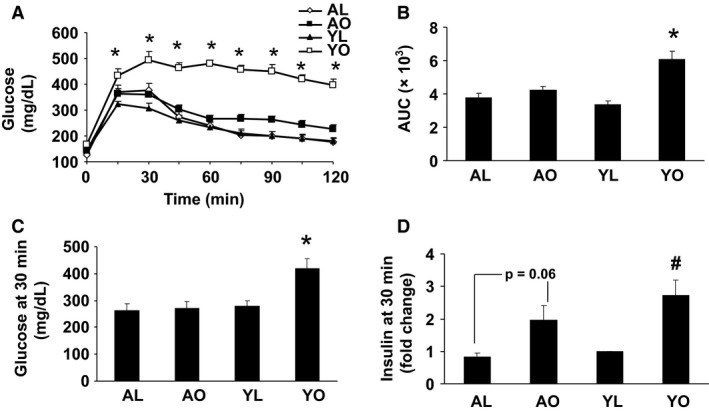
Glucose tolerance tests and insulin response to a glucose load. Glucose tolerance tests were performed as described in Materials and Methods. Briefly, mice were injected with 1.5 g/kg glucose and blood glucose concentration determined from tail vein blood samples every 15 min for 135 min. Panels A and B show the glucose curves and the area under the glucose curve (AUC), respectively. Panels C and D show the 30‐min blood glucose and insulin concentrations from a separate experiment following IP glucose injection. Results are presented as the means ± SE for a minimum of six animals/group analyzed individually. Significant differences are indicated (*as compared to the other three groups, ^#^as compared to AL and YL, *P* < 0.05).

AO and YO had similar body weight and total adiposity, whereas AL mice were somewhat heavier and had a higher total fat mass as compared to the YL mice (Fig. 1, Panels A and C). However, the combined gonadal, perirenal, and mesenteric fat depots were somewhat greater in AO compared to YO (4.9 ± 0.1 g vs. 3.8 ± 0.3 g, *P* < 0.05), a difference that was also present in AL compared to YL (2.2 ± 0.2 g vs. 1.4 ± 0.2 g, *P* < 0.05). Lean mass was higher in aged animals as compared to young animals (Fig. 1B). Liver TG content was significantly higher in YO and AO compared to lean groups (AL and YL, Fig. 1D), but was substantially higher in YO compared to AO (*P* < 0.05). YL had higher energy expenditure than obese groups, but not AL (Fig. 1E, *P* < 0.05). YO were more sedentary than the other three groups of animals (*P* < 0.05), all of which had similar activity levels (Fig. 1F). YO were substantially more glucose intolerant and insulin resistant than all other groups, as evidenced by plasma glucose concentrations that remained elevated throughout the 135‐min sampling period of a GTT, and a greater insulin response (Fig. 2, Panels A–C). Interestingly, AO had similar glucose tolerance to both groups of lean mice. However, the plasma insulin concentration in AO was higher than AL (*P* < 0.06) at peak plasma glucose concentration, indicating that AO were more insulin resistant than AL (Fig. 2D) Thus, YO were more insulin resistant than AO, despite a similar body weight and degree of adiposity.

### Dendritic cell and macrophage infiltration of AT and liver is unaltered by age or increased adiposity in isolation from a HFD

**Figure 3:**
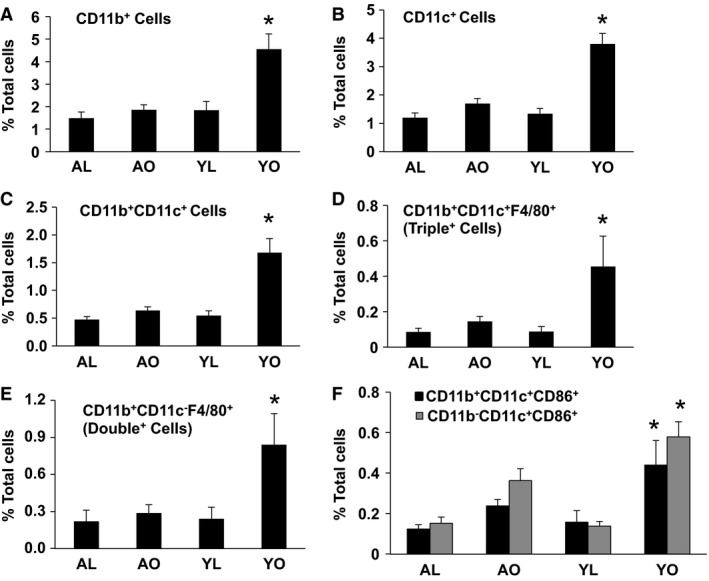
The influence of aging, obesity, and diet on CD11b^+^ and CD11c^+^ cells and their subpopulations in adipose tissue (AT). Stromal vascular cells were isolated from AT (epididymal and perirenal) of AL, AO, YL, and YO mice, stained for the CD45, CD11b, CD11c, CD86, and F4/80 markers and analyzed by flow cytometry. Data are presented as mean ± SE (minimum of 4–6 animals/group analyzed individually). Significant differences are indicated (*as compared to other three groups, *P* < 0.05).

Since inflammation, and more specifically, immune cell infiltration of AT and liver, has been implicated in the pathogenesis of the metabolic abnormalities of obesity, we set out to establish if dissimilar immunophenotype responses to diet and aging‐induced obesity could explain the different metabolic outcomes in these two models. AT of YO had substantially higher macrophage and dendritic cells (CD11b^+^ and CD11c^+^ markers, *P* < 0.05) content compared to YL (Fig. 3, Panels A and B). Importantly, these increases were not apparent in AO. Indeed, these populations in AO were similar to levels observed in AL mice, which in turn were similar to YL, demonstrating that diet, and not degree of adiposity or aging, was the main determinant of CD11b^+^ and CD11c^+^ cell infiltration in AT in this study. Further analysis of macrophage/dendritic cell subpopulations revealed increased (*P* < 0.05) infiltration of the well‐described obesity lineage CD11b^+^ CD11c^+^ F4/80^+^ (triple^+^), CD11b^+^ CD11c^+^, and double positive (CD11b^+^ CD11c^−^ F4/80^+^) populations in the AT of YO mice, but not in AT of AL or AO (Fig. 3, Panels C–E). Finally, activated subpopulations of CD11c^+^ cells (CD11b^+^ CD11c^+^ CD86^+^, CD11b^−^ CD11c^+^ CD86^+^) were elevated in the YO mice, but not the other groups (Fig. 3F, *P* < 0.05).

**Figure 4:**
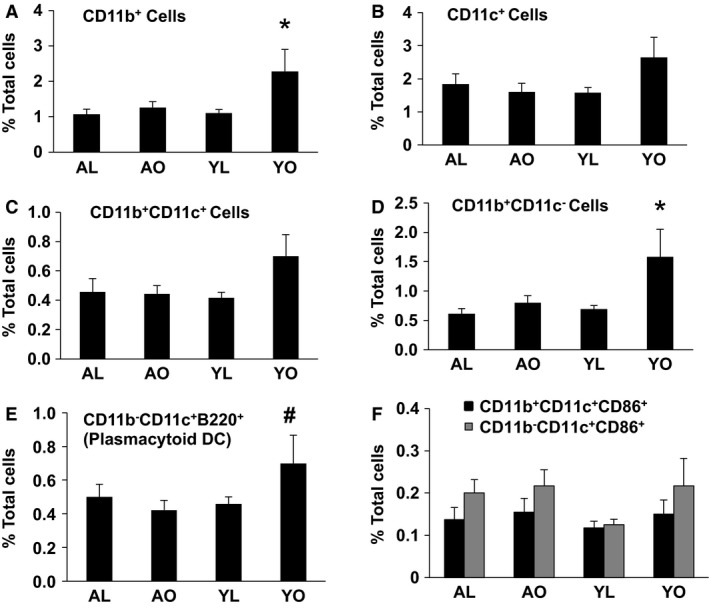
The influence of aging, obesity, and diet on CD11b^+^ and CD11c^+^ cells and their subpopulations in the liver. Liver immune cells were isolated and stained for the CD45, CD11b, CD11c, CD86, and B220 markers and analyzed by flow cytometry. Data are presented as mean ± SE (minimum number of four animals/group analyzed individually). Significant differences are indicated (*as compared to other three groups, ^#^as compared to AO and YL groups, *P* < 0.05).

In the liver, a similar immunophenotype pattern to that observed in AT was present (Fig. 4, Panels A–F), although increases in some cases did not reach significance. Notably, plasmacytoid dendritic cells, which we have recently described as the predominant CD11c^+^ population that increases in liver in diet‐induced obesity (Stefanovic‐Racic et al. 2012), was highly elevated in YO (*P* < 0.05), but was unaltered in AO (Fig. 4, Panel E). These data, again, emphasize that it is diet, as opposed to obesity per se, that is the main driving force of macrophage and dendritic cell infiltration of AT and liver in this study. Finally, there were no effects of either diet‐ or aging‐induced obesity on the myeloid cell pattern in spleen (Figure S1) or mesenteric tissue (data not shown).

### T‐cell infiltration of AT is influenced by age predominantly

**Figure 5:**
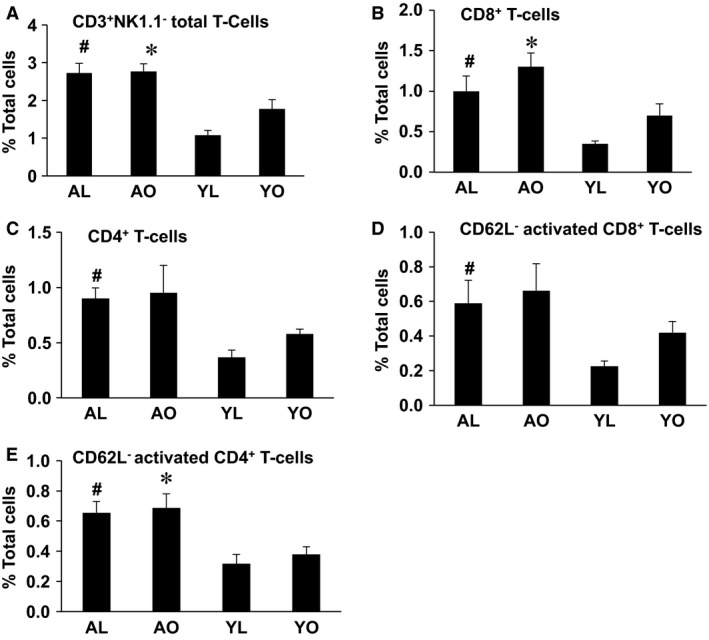
The influence of aging, obesity, and diet on CD3^+^NK1.1^−^ cells (T cells) and their subpopulations in adipose tissue (AT). Stromal vascular cells were isolated from AT (epididymal and perirenal) of AL, AO, YL, and YO mice, stained for CD3, NK1.1, CD4, CD8, and CD62L markers and analyzed by flow cytometry. Data are presented as mean ± SE for a minimum of five animals/group analyzed individually. Significant differences are indicated (^#^as compared to YL, *as compared to YO, *P* < 0.05).

Lymphoid cell infiltration, specifically T cells, has also been implicated in the inflammation and metabolic abnormalities of obesity. Thus, we next assessed T cell populations in the AT and liver. AT of AL had substantial increases in total T cells (CD3^+^ NK1.1^−^), helper T cells (CD3^+^ CD4^+^), and effector (CD3^+^ CD8^+^) T cells compared to YL (all *P* < 0.05), and these increases were not further enhanced in AO (Fig. 5, Panels A–C). Furthermore, activated CD3^+^ CD4^+^ and CD3^+^ CD8^+^ T cells (CD62L^−^) were elevated in AL compared to YL (all *P* < 0.05), and again were not further elevated in AO (Fig. 5, Panels D and E). Total T cells and activated CD3^+^ CD4^+^ and CD3^+^ CD8^+^ T cells were also increased in AO compared to YO. On the contrary, NKT cells (CD3^+^ NK1.1^+^) were decreased in the AL and AO mice compared to YO and YL (data not shown, *P* < 0.05). There were no differences in B cells and NK cells (CD3^−^ NK1.1^+^) between all four groups (data not shown), consistent with a specific effect of aging on T‐cell populations in AT. Finally, there were no alterations in spleen total T cells (CD3^+^ NK1.1^−^), helper T cells (CD3^+^ CD4^+^), and effector (CD3^+^ CD8^+^) T cells in aged compared to young mice, with the one exception that AO mice had higher CD62L^+^ CD8 cells (Figure S2, *P* < 0.05). Together, these data demonstrate that aging per se alters the lymphocyte profile of AT. Furthermore, since AL and YL display similar insulin sensitivity, the data demonstrate that increases in AT T cells alone is insufficient to induce this metabolic abnormality, at least in the context of aging.

**Figure 6:**
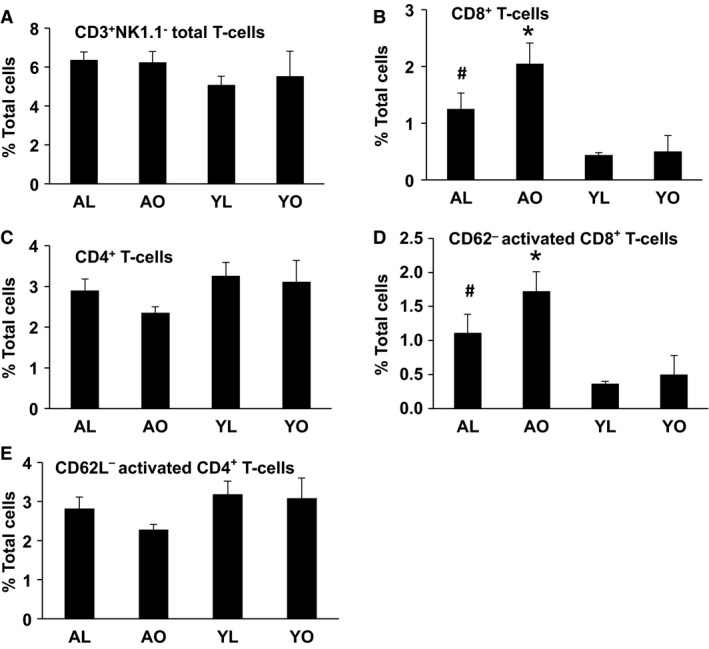
The influence of aging, obesity, and diet on CD3^+^NK1.1^−^ cells (T cells) and their subpopulations in liver. Liver immune cells were isolated and stained for CD3, NK1.1, CD4, CD8, and CD62L markers and analyzed by flow cytometry. Data are presented as mean ± SE (minimum of five animals/group analyzed individually). Significant differences are indicated (^#^compared to YL, *compared to YO, *P* < 0.05).

An evaluation of the liver produced an interesting, albeit more nuanced, pattern of lymphoid cell infiltration. Thus, liver total T cells (CD3^+^ NK1.1^−^) and helper T cells (CD3^+^ CD4^+^) were unaltered by aging (Fig. 6, Panels A and C). However, effector (CD3^+^ CD8^+^) T cells were increased in aged cohorts compared to young cohorts and, furthermore, activated CD3^+^ CD8^+^ T cells were increased (Fig. 6, Panels B and D, *P* < 0.05). Finally, there was an indication that obesity in aging further increased effector T cells, but these increases did not reach significance (*P* > 0.05). In total, these data again emphasize an effect of aging per se on lymphocyte populations, with a potentially minor effect of obesity.

### Immune cells from the liver of age associated obesity mice are less proinflammatory compared to diet‐induced obesity

**Figure 7:**
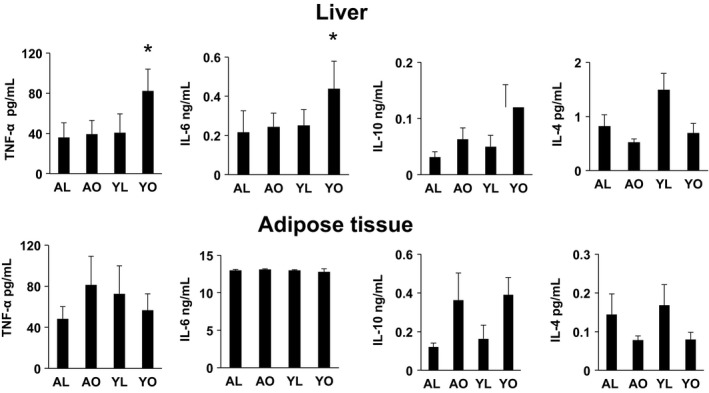
Cytokine production by liver immune cells and adipose tissue (AT) stromal vascular cells (SVC). Isolated immune cells from liver (upper panel) and SVC from AT (lower panel) were cultured for 18 h in complete DMEM medium. The harvested media was analyzed for the indicated cytokines using Luminex. Data are presented as mean ± SE (minimum of five animals/group analyzed individually). Significant differences are indicated (*P < 0.05).

Reduced infiltration of immune cells into tissues in AO likely contributes to the better metabolic regulation compared to YO. We investigated this issue further by addressing the effects of aging‐associated obesity and diet‐induced obesity on the Th‐1/Th‐2 cytokine profile of the SVC of AT and the liver immune cell fraction (CD45^+^). Media from liver immune cells from YO cultured for 18 h showed a substantial increase in the Th‐1 cytokines TNF‐α and IL‐6 compared to AO (Fig. 7, top panel, *P* < 0.05). Surprisingly, these differences were not present in media from the incubation of the SVC of AT from YO and AO (Fig. 7, bottom panel). Of note, production of the Th‐2 cytokines IL‐10 and IL‐4 from liver immune cells of YO were also increased compared to AO, although these increases did not reach statistical significance (*P* > 0.05). As for Th‐1 cytokines, Th‐2 cytokine production from the SVF of AT was similar in YO and AO. Together, these data suggest that despite similar body weight and adiposity, a HFD promotes a stronger proinflammatory cytokine response in the liver compared to the effects of aging.

## Discussion

Obesity has become a major worldwide health problem, increasing in all age groups including older adults (>65 years of age), and is associated with an increased risk of developing diseases associated with the metabolic syndrome such as type 2 diabetes, hypertension, and nonalcoholic fatty liver disease (NAFLD). While there is a proportion of aging‐associated obesity that may be related to loss of muscle mass, reduced activity, and/or changes in metabolic rate, diet is also a major contributor to this “obesity epidemic.” In this study, we explored the metabolic and immunological differences between the obesity of aging and obesity induced by diet. Our data demonstrate that diet‐induced obesity has a more adverse metabolic outcome compared to aging‐associated obesity, presenting as more severe insulin resistance and liver steatosis. Notably, the more benign metabolic phenotype of aging is associated with an AT and liver immunophenotype that does not resemble the “typical” obesity‐associated immune cell infiltration and Th‐1 polarization pattern of AT and liver.

The two groups of obese animals (“young” and “old”) used in this study were matched to have similar body compositions. Our expectation was that the similar levels of obesity would result in comparable metabolic dysfunction. However, as our data demonstrate, this was not the case. Indeed, the obesity of aging, while resulting in a modest increase in liver triglyceride, had no impact on glucose tolerance when compared to an aged matched lean cohort, or the lean young mice. This was a somewhat unexpected finding, as it suggests that the metabolic consequences of obesity are, to some degree, dependent on the pathway taken to the development of obesity. Thus, in the current case, it appears that it is not the weight gain alone, but the rapidity of weight change and/or the contribution of excess nutrients to the weight gain that explains the adverse metabolic outcomes. Weight gain in AO mice occurred over a prolonged period of time while being maintained on the low fat diet, unlike YO animals, which gained a similar amount of weight in a much shorter period of time, while on the HFD (throughout life in AO vs. 14 weeks in YO, respectively). Thus, aged mice appear to have metabolically “adapted” to the increased adiposity, thereby explaining the differences observed between the two groups. There are striking parallels here to the phenotype of the obese, but metabolically healthy, human populations that have been described previously (Karelis et al. 2005; Primeau et al. 2011) and to some genetically obese mouse models that maintain metabolic health despite their obesity (Patsouris et al. 2008; de Roos et al. 2009; Spite et al. 2011; Wang et al. 2013). In each case, it appears that the “quality” of the obesity is an important determinant of the metabolic outcome. Another implication of our findings is that excessive weight gain starting at a younger age in humans may be more metabolically detrimental compared to development of obesity later in life. This is supported by several recent studies showing that early onset of obesity in childhood leads to more detrimental cardiometabolic consequences than weight gain starting later in life (Freedman et al. 2001; Boney et al. 2005; Holterman et al. 2013; Ogden et al. 2014). Similar to our study, it is likely that the excessive weight gain of childhood is partially a consequence of environment (i.e., excess calories/“bad” calories) while that of aging is influenced to a greater degree by altered physiological variables (i.e., decreased metabolic rate, loss of muscle mass, decreased activity).

Numerous studies have shown increased infiltration of macrophages and dendritic cells into AT and liver in diet‐induced obesity in rodents and human obesity (Weisberg et al. 2003; Xu et al. 2003; Huang et al. 2010; Olefsky and Glass 2010; Wentworth et al. 2010; Gregor and Hotamisligil 2011; Lumeng and Saltiel 2011; Osborn and Olefsky 2012; Stefanovic‐Racic et al. 2012). As these cells are proposed to be a primary source of obesity‐induced inflammation and a major player in the development of IR, we sought to determine whether the observed metabolic differences between YO and AO mice were related to differences in the degree of tissue immune cell infiltration and cytokine production. Indeed, this proved to be the case. As expected, the metabolic dysfunction in diet‐induced obese mice correlated well with increased AT infiltration of macrophages and dendritic cells (CD11b^+^ cells and CD11c^+^ cells and their subpopulations). By contrast, aging‐induced obesity displayed no such infiltration, which again correlates well with the metabolic phenotype of these mice. Furthermore, the TNF‐α and IL‐6 production of liver immune cells from YO mice were, as expected, elevated, and these increases were absent in old obese mice. Surprisingly, we did not observe these increases in SVC from diet‐induced obese AT, for unclear reasons. These data reiterate the concept that increased adiposity per se does not result in immune cell infiltration of AT, and suggest that the physiological/pathophysiological processes that occur with rapid adipose accumulation induced by the high fat (i.e., cellular stress, hypoxia, rapid tissue remodeling) are the driving force for immune cell infiltration as has been proposed by others. The data are also consistent with studies in mice that demonstrate that the prevention of infiltration and/or Th‐1 polarization of immune cells prevents the development of insulin resistance, despite increased adiposity (Kanda et al. 2006; Odegaard et al. 2007).

There were no significant differences in the macrophage and dendritic cell content or polarization in AT and liver of AL and YL mice, suggesting that aging per se does not impact the tissue content of these cells. However, aging was associated with an AT and liver immunophenotype characterized by a marked elevation of the lymphoid cell populations, specifically CD8^+^ and CD4^+^ T cells, and a majority of these cells were activated (as assessed by altered CD62L expression) compared to YL or YO mice. These increases were not exacerbated by obesity in aging, re‐emphasizing that the effect is aging related. These data are consistent with the work of Lumeng et al., which demonstrated that aging is associated with an increase in both helper and effector T cells in AT (Lumeng et al. 2011). Drawing on their data, at least some of the activated CD4^+^ T cells observed in our study may be regulatory T cells; however we did not include a Foxp3 analysis in our experiments, which would have allowed us to confirm this possibility. While previous studies have implicated elevated T cells in the pathogenesis of metabolic dysregulation in obesity (Nishimura et al. 2009), there was no clear difference in insulin sensitivity between the AL and YL mice in the current study, despite observing T cell increases in aged mice. These data suggest that, at least in the context of aging, elevations in tissue T‐cell content alone do not induce metabolic dysfunction associated with obesity.

Although AO mice had significantly lower liver triglyceride content compared to YO mice, the level in AO mice was still higher than in the lean aged animals. However, there were no discernible immunophenotypic differences in AT and liver between AO and AL mice, suggesting that the mild hepatic steatosis noted in the AO mice is not associated with chronic inflammation. Here again, there are differences to diet‐induced obesity, where liver inflammation has been associated with the development of liver steatosis (Rector et al. 2008; Fabbrini et al. 2010; Huang et al. 2010; Tilg and Moschen 2010; Wentworth et al. 2010). Indeed, liver steatosis can be prevented or improved in rodents on HFD, when inflammation is prevented (Huang et al. 2010). Our data in aged mice are more similar to observations in elderly human subjects with NAFLD, in whom elevated liver TG content is less likely to be associated with an increased risk of cardiovascular disease or metabolic syndrome (Koehler et al. 2012).

In summary, our study highlights important immunophenotypic and metabolic divergences between two models of obesity induced by different environmental processes, aging or diet. The data clearly demonstrate a substantially greater negative metabolic impact of diet‐induced obesity compared to aging‐induced obesity. These differences are closely correlated with the absence of macrophage and dendritic cell infiltration of AT and liver in aging‐induced obesity. We speculate that the earlier onset, in addition to the rapidity, of adiposity accumulation, and/or exposure to excess “toxic” nutritional components in diet‐induced obesity induces a substantially greater physiological stress compared to aging‐associated obesity, which in turn induces a robust inflammatory response. These observations may be relevant to the rapid increase in the prevalence of obesity in the child and adolescent populations, linked with increased consumption of a high‐fat/high‐sugar diet starting at a relatively early age (Nicklas et al. 2003). Our data would suggest that the metabolic outcomes in this population may be more detrimental then in the more gradual attainment of obesity observed in older human populations.

## Conflict of Interest

None declared.

## Supporting information




**Figure S1.** Myeloid cell content of the spleen.

**Figure S2.** T‐cell content of the spleen.
