# Supplementary figures and images for: Similar degrees of obesity induced by diet or aging cause strikingly different immunologic and metabolic outcomes
Source: Physiol Rep. 2016 Mar 31;4(6):e12708. doi: 10.14814/phy2.12708 (PMC4814885; doi:10.14814/phy2.12708)

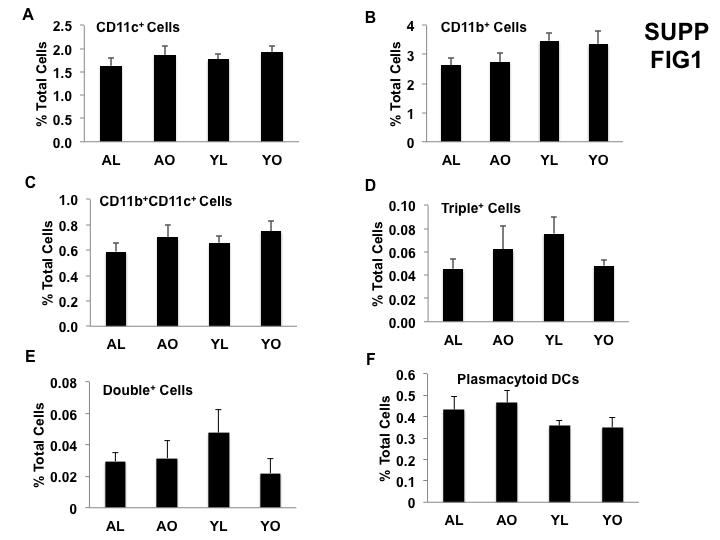

Supplement: Supplementary file 1 — Figure S1. Myeloid cell content of the spleen. [file PHY2-4-e12708-s001.tiff]

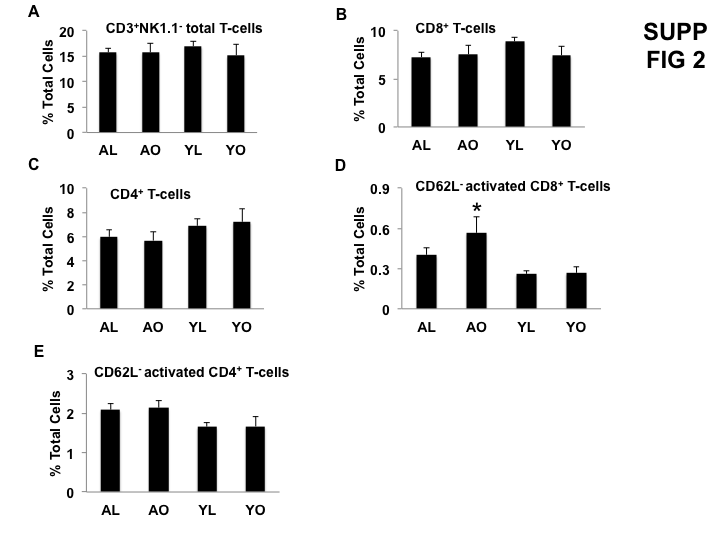

Supplement: Supplementary file 2 — Figure S2. T‐cell content of the spleen. [file PHY2-4-e12708-s002.tiff]
